# Geographical location, cigarette risk perceptions, and current smoking among older US adults

**DOI:** 10.18332/tid/191827

**Published:** 2024-09-12

**Authors:** Jenny E. Ozga, Cassandra A. Stanton, James D. Sargent, Alexander W. Steinberg, Zhiqun Tang, Laura M. Paulin

**Affiliations:** 1 Westat Behavioral Health and Health Policy Practice, Rockville, United States; 2 Department of Pediatrics, Geisel School of Medicine, Hanover, United States; 3 Department of Biomedical Data Science, Geisel School of Medicine, Hanover, United States; 4 Department of Pulmonary and Critical Care Medicine, Dartmouth Hitchcock Medical Center, Lebanon, United States

**Keywords:** rural, harm perception, communication, culture, social norms

## Abstract

**INTRODUCTION:**

Cigarette smoking and smoking-related lung disease are more common in rural (vs urban) areas of the United States (US). This study examined relationships between geographical location, cigarette risk perceptions, and current smoking among older adults who are at greatest risk of developing smoking-related lung disease.

**METHODS:**

The study was a secondary data analysis of 12126 respondents aged ≥40 years from Wave 5 of the Population Assessment of Tobacco and Health Study. Weighted descriptive statistics and Poisson regressions assessed current smoking (vs never or former) as a function of geographical location in a stepwise fashion, first unadjusted, then adjusting for sociodemographic characteristics, and finally for both sociodemographic characteristics and cigarette risk perceptions (4-item scale), in three separate models. Sensitivity analyses examined whether individual risk perceptions items had a greater impact on the association between geographical location and current smoking.

**RESULTS:**

Current smoking was more common among rural (20.6%) than urban (17.6%) residents. The risk ratio (RR) for rural (vs urban) residence on current smoking decreased from 1.17 (95% CI: 1.03–1.32) to 1.14 (95% CI: 1.01–1.29) to 1.08 (95% CI: 0.96–1.21) across the stepwise models. Lower cigarette risk perceptions confounded the rural-current smoking association and was an independent risk factor for smoking (adjusted RR, ARR=2.15; 95% CI: 1.94–2.18). In sensitivity analyses, believing that cigarettes are very or extremely (vs somewhat, slightly, or not at all) harmful to health and agreeing (vs not agreeing) that secondhand smoke causes lung disease in people who do not smoke, confounded the rural-current smoking association whereas beliefs about smoking causing lung cancer or lung disease in people who smoke did not.

**CONCLUSIONS:**

Lower cigarette risk perceptions among rural residents confounded the positive association between rural residence and current smoking. Results from sensitivity analyses highlight potential targets for communication campaigns aimed at promoting more accurate perceptions of the harmful health consequences of cigarette smoking.

## INTRODUCTION

Despite an overall decline in cigarette smoking over the past several years, smoking remains the leading cause of preventable death and disease in the United States (US)^1,2^. Rates of cigarette smoking^3-5^, smoking-related lung disease^6,7^, and associated morbidity and mortality^2,8,9^ are higher in non-urban (vs urban) areas of the US, independent of differences in socioeconomic status. National cross-sectional US survey data suggest that the disparities between urban and rural cigarette smoking^3,4^ and related lung disease^6^ have been increasing over time. According to data from the National Health Interview Survey, in 2022, 18.1% of US adults living in rural communities reported smoking cigarettes compared to 10.5% of adults in urban communities^10^. Among those who smoke, adults in rural (vs urban) communities smoke more cigarettes per day^10^ and started smoking at a younger age^11^, contributing to greater nicotine dependence and more difficulties with cigarette cessation^12^. The increased rate of smoking is one of the multiple intersecting factors that have led the US Food and Drug Administration’s Center for Tobacco Products (FDA CTP) to classify rural Americans as a ‘population of special relevance’^13,14^.

Perceiving cigarettes as less (vs more) harmful^15-17^ has been associated with smoking among both youth and adults. Less is known about the relationship between risk perception and smoking among older adults, or particularly among older adults in rural areas, who are at greatest risk of developing smoking-related lung disease. In one study using data from a national US longitudinal cohort study, older adults (aged ≥65 years) who smoked cigarettes perceived cigarettes as less harmful than younger adults (aged 18–24 years)^18^. However, this study did not assess differences in harm perceptions and cigarette smoking based on geographical location. Cigarette smoking risk perceptions can be shaped by communication campaigns aimed at reducing tobacco use (e.g. The Real Cost campaign)^19^ and encouraging smoking cessation (e.g. Tips®)^20^. Determining whether older adults living in urban and rural areas of the US differ in cigarette-related risk perceptions could inform important targets for such campaigns.

The current secondary data analysis used data from a nationally representative US sample of older (aged ≥40 years) adults to examine cross-sectional relationships between geographical location, cigarette risk perceptions, and current smoking. Separate regression models were conducted in a stepwise fashion to determine whether the association between geographical location and current smoking would be significantly impacted by adjusting for important sociodemographic covariates (including income and education) and cigarette risk perceptions.

**Figure 1:**
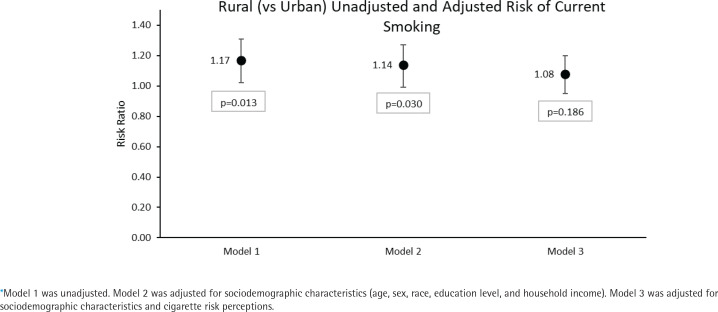
Estimated risk ratios for the association between rural (vs urban) geographical location and current (vs never or former) cigarette smoking among adults aged ≥40 years at Wave 5 (2018–2019) of the Population Assessment of Tobacco and Health (PATH) Study in three stepwise models*

## METHODS

### Study design and sample

We conducted a cross-sectional secondary data analysis using data from Wave (W) 5 of the Population Assessment of Tobacco and Health (PATH) Study, a national longitudinal cohort survey of US youth and adults. Data were collected in respondents’ households using computer-assisted self-interviews administered in English or Spanish, as appropriate. Survey data were collected from adults (aged ≥18 years) in 2013–2014 (W1), 2014–2015 (W2), 2015–2016 (W3), 2016–2017 (W4), and 2018–2019 (W5). Further details about the PATH Study design, methods, and instruments are publicly available^21^. The current secondary data analysis used the W5 Adult Restricted Use Files^21^ and was limited to adults aged ≥40 years at W5 (n=12126). See Supplementary file Figure 1 for a flow diagram of the study sample. This study qualified as exempt per guidelines of the Westat Institutional Review Board and the Dartmouth Health Human Research Protection Program.

### Measures

*Current cigarette smoking*

The primary outcome was current (vs never/former) cigarette smoking at W5. We used the PATH Study-derived variables for never, current established, and former established cigarette smoking to create a two-category smoking status variable capturing never/former (coded 0) or current (coded 1) smoking. Current established cigarette smoking was defined as smoking at least 100 cigarettes in lifetime and currently smoking every day or some days. Former established cigarette smoking was defined as smoking at least 100 cigarettes in lifetime and not smoking in the past 12 months or currently smoking not at all. Never smoking was defined as smoking fewer than 100 cigarettes in lifetime.

*Geographical location*

The primary exposure variable was geographical location (released in July 2024 in the PATH Study Restricted Use Files)^21^, which was categorized as urban, suburban, town, or rural. Categories were assigned to individual respondents at W5 at the US Census block level using 2021 Census block definitions. Respondents’ geolocations were coded to match 12-level locale classifications prior to being collapsed into the four categories listed^21^. Additional information can be found at: https://nces.ed.gov/programs/edge/Geographic/LocaleBoundaries^22^.

*Cigarette risk perceptions*

Cigarette risk perceptions at W5 were assessed through four separate survey items. Three of the items were related to beliefs about the health risks of cigarette smoking: ‘Based on what you believe, how much do you agree or disagree with the following statements? Smoking can cause: a) lung cancer in smokers, b) lung disease (such as emphysema) in smokers, and c) lung disease in non-smokers from secondhand smoke’. Response options for all three of these items ranged from 1 (Strongly agree) to 5 (Strongly disagree). The fourth item (d) was more general: ‘How harmful do you think cigarettes are to health?’, with response options ranging from 1 (Not at all harmful) to 5 (Extremely harmful). The four individual cigarette harm perception items were combined into a 4-item scale (Cronbach’s alpha=0.78). To create the scale, items were reverse coded as necessary such that across all four items, higher values indicated lower perceptions of risk. Then, an average risk perception score across the four items was calculated for each respondent (ranging from 1 to 5).

After examining the 4-item scale in a primary multivariable regression model (Model 3), we examined each individual risk perception item in four secondary separate models (Models 3a–3d) during sensitivity analyses to determine which element(s) had the strongest association with current smoking and which impacted the geographical location association most. For sensitivity analyses, response options for each item were dichotomized into: ‘Agree’ (strongly agree/agree, coded as 0) versus ‘Not agree’ (neither/disagree/strongly disagree, coded as 1) or ‘Harmful’ (very/extremely, coded as 0) versus ‘Not harmful’ (slightly/somewhat/not at all, coded as 1) as appropriate.

*Sociodemographic covariates*

Sociodemographic covariates were categorized as shown in Table 1. From W5, these included age, sex, race, education level, and total household income in the past 12 months. Missing data on age, sex, and race were imputed as described in the PATH Study Restricted Use Files User Guide^21^.

**Table 1:** Sociodemographic and cigarette use characteristics overall and by geographical location among adults aged ≥40 years at Wave 5 (2018–2019) of the Population Assessment of Tobacco and Health (PATH) Study (N=12126)

	*Overall (N=12126)*	*Urban (N=3635)*	*Suburban (N=4214)*	*Town (N=1188)*	*Rural (N=3089)*
	*Weighted column % (95% CI)*				
**Age** (years)					
40–49	25.2 (24.3–26.0)	27.8 (26.2–29.5)	27.0 (25.5–28.6)	22.0 (18.7–25.6)	20.3 (18.5–22.3)
50–59	26.9 (26.1–27.7)	26.2 (24.5–28.0)	28.0 (26.5–29.6)	23.9 (21.4–26.5)	27.2 (25.4–29.2)
60–69	25.0 (24.1–26.0)	24.6 (22.7–26.7)	22.3 (20.7–23.9)	25.2 (22.1–28.6)	29.7 (27.2–32.4)
70–79	16.0 (15.2–16.8)	14.4 (13.0–15.9)	16.3 (14.8–17.9)	17.6 (14.9–20.6)	16.8 (15.1–18.7)
≥80	6.9 (6.3–7.6)	6.9 (5.8–8.3)	6.4 (5.2–7.9)	11.3 (9.2–13.8)	5.9 (4.7–7.3)
**Sex**					
Male	46.7 (46.2–47.1)	44.4 (42.7–46.2)	46.4 (44.9–47.9)	43.7 (40.4–47.0)	50.8 (48.5–53.2)
Female	53.3 (52.9–53.8)	55.6 (53.8–57.3)	53.6 (52.1–55.1)	56.3 (53.0–59.6)	49.2 (46.8–51.5)
**Race**					
White	80.1 (79.4–80.8)	70.9 (68.2–73.3)	78.8 (76.4–81.0)	87.6 (83.6–90.8)	89.9 (87.0–92.1)
Black	11.4 (11.1–11.7)	18.7 (16.7–21.0)	10.2 (8.6–11.9)	7.8 (5.2–11.6)	6.4 (4.4–9.2)
Other/Multiple	8.5 (8.0–9.1)	10.5 (8.9–12.2)	11.1 (9.6–12.8)	4.5 (3.1–6.6)	3.8 (2.9–4.9)
**Education level**					
<High school or GED	16.6 (16.1–17.1)	20.0 (18.2–22.0)	12.9 (11.6–14.3)	19.2 (16.5–22.3)	17.4 (15.7–19.3)
High school degree	23.0 (22.4–23.6)	18.0 (16.2–20.0)	20.1 (18.5–21.8)	31.6 (27.9–35.6)	30.0 (28.1–32.1)
Some college or Associate’s degree	28.8 (28.2–29.4)	27.1 (25.4–28.8)	28.7 (27.4–30.1)	30.1 (26.8–33.7)	30.6 (28.7–32.5)
Bachelor’s or advanced degree	31.5 (31.1–32.0)	34.9 (32.8–37.0)	38.3 (36.0–40.6)	19.0 (16.2–22.2)	21.9 (19.9–24.1)
**Household income ($)**					
≥100000	22.7 (21.7–23.8)	22.4 (20.1–24.8)	28.6 (26.4–31.0)	11.7 (8.8–15.4)	18.0 (15.9–20.4)
50000–99999	24.7 (23.6–26.0)	20.6 (18.7–22.7)	26.2 (24.1–28.3)	24.8 (21.2–28.8)	27.3 (24.5–30.2)
25000–49999	20.1 (19.1–21.1)	20.2 (18.2–22.3)	17.4 (15.8–19.0)	23.1 (20.6–25.7)	23.2 (20.8–25.8)
<25000	24.7 (23.9–25.6)	30.3 (28.0–32.6)	18.6 (17.2–20.1)	33.1 (28.1–38.6)	24.7 (22.4–27.0)
**Cigarette smoking status**					
Never	48.6 (47.0–50.3)	50.6 (48.3–53.0)	50.2 (47.4–53.1)	47.0 (41.2–52.9)	44.4 (41.3–47.6)
Former	33.9 (34.5–35.5)	31.8 (29.6–34.0)	35.1 (32.7–37.7)	32.8 (27.9–38.0)	35.0 (32.4–37.7)
Current	17.4 (16.7–18.2)	17.6 (16.3–19.0)	14.6 (13.6–15.7)	20.2 (17.6–23.1)	20.6 (18.6–22.7)

### Statistical analysis

Weighted descriptive statistics and Poisson regressions were used to evaluate associations between geographical location, cigarette risk perceptions, and current smoking^23^. Given that current smoking is not a rare outcome, we chose to use Poisson regression with more easily interpreted risk ratios (vs logistic regressions with odds ratios) to generate conservative estimates for associations between geographical location and current smoking^22^. Unadjusted models and models adjusted for age, sex, race, education level, and household income were examined to estimate risk ratios (RRs) and adjusted RRs (ARRs). Separate regression models were conducted in a stepwise fashion. Model 1 examined the unadjusted association between geographical location and current smoking. Model 2 examined this same association adjusted for sociodemographic covariates. Model 3 examined ARRs for geographical location and mean cigarette risk perceptions (using the 4-item scale), also adjusted for sociodemographic covariates. Stepwise models were built based on previous empirical findings. Specifically, prior work has shown that age, gender, race, education level, and income are all independently associated with current smoking when concurrently present in a multivariable model ^23^. Therefore, after examining the unadjusted association between geographical location and current smoking (Model 1), these known sociodemographic covariates were entered into an additional step (Model 2), prior to examining how accounting for cigarette risk perceptions (Model 3) would impact the association between geographical location and current smoking above and beyond covariates known to be associated with current smoking.

Respondents who had valid data on the variables used in specific regression models were included in the models. Respondents who were missing data on one or more of the variables in a specific regression model were removed from the respective model. All analyses were weighted using the W5 single-wave survey weights for the W4 cohort, which included full sample and 100 replicate weights, to produce nationally representative estimates. The weights employed in our analysis accounted for attrition and non-response bias, as well as replenishment of the sample at W4, in order to obtain estimates for the US population^21^. Variances were computed using the balanced repeated replication (BRR) method^24^ with Fay’s adjustment set to 0.3 to increase estimate stability^25^ using the following code in Stata: svyset [pweight=R05_A_S04WGT], brr(R05_A_S04WGT1 – R05_A_S04WGT100) vce(brr) mse fay(0.3). Extensive details on how replicate weights were constructed by the PATH Study can be found in the PATH Study Restricted Files User Guide^21^. All analyses were conducted using Stata/MP 17.0 (www.stata.com/statamp/).

*Sensitivity analysis*

Four additional multivariable models (Models 3a–3d) examined ARRs for geographical location and each individual risk perception item from the 4-item scale to determine whether certain scale element(s) had a greater impact on the association between geographical location and current smoking. For sensitivity analyses, response options for each item were dichotomized into ‘Agree’ (coded 0) vs ‘Not agree’ (coded 1) or ‘Harmful’ (coded 0) vs ‘Not harmful’ (coded 1), as appropriate.

## RESULTS

### Sample characteristics

Table 1 shows sociodemographic characteristics overall and by geographical location. Approximately one-third (30.0%) of the analytic sample were located in urban areas, 34.8% were in suburban areas, 9.8% were in town areas, and 25.5% were in rural areas. Participants were mainly female (53.3%), of White race (80.1%), and the mean age was 59.65 years (SD=12.30). There was a relatively even distribution of respondents across total household income levels and age categories. When comparing respondents across geographical locations, rural (vs urban) areas had a higher proportion of males (50.8% vs 44.4%) and White residents (89.9% vs 70.9%) and lower proportions of Black (6.4% vs 18.7%) or Other/Multiple race residents (3.8% vs 10.5%). Rural areas also had more respondents aged ≥60 years whereas urban areas had more respondents aged 40–49 years. Rural (vs urban) areas had fewer residents who reported an annual household income of ≥$100000 (18.0% vs 22.4%) and fewer residents who reported having a Bachelor’s or advanced degree (21.9% vs 34.9%). Respondents from urban and suburban areas were similar across sociodemographic characteristics, whereas respondents from rural and town areas were similar.

### Geographical location, cigarette risk perceptions, and current smoking

Current smoking was more common among rural (20.6%) and town (20.2%) versus urban (17.6%) and suburban (14.6%) residents (Table 1). Shown in Table 2, in the unadjusted model (Model 1), rural (vs urban) residence was significantly positively associated with current (vs never/former) smoking (RR=1.17; 95% CI: 1.03–1.32) whereas suburban (vs urban) residence was significantly negatively associated with current smoking (RR=0.83; 95% CI: 0.74–0.92). The RR for rural (vs urban) residence when the model was adjusted for sociodemographic covariates (Model 2) decreased but remained statistically significant (ARR=1.14; 95% CI: 1.01–1.29) while the RR for suburban (vs urban) residence became non-significant. Further adjustment for cigarette risk perceptions (Model 3) confounded the association for rural residence (ARR=1.08; 95% CI: 0.96–1.21). Lower cigarette risk perceptions (Model 3) were also associated with significantly increased risk of current cigarette smoking, independent of geographical location and sociodemographic covariates (ARR=2.05; 95% CI: 1.94–2.18).

**Table 2:** Primary models: stepwise bivariable and multivariable regressions examining associations between geographical location, cigarette risk perceptions, and current (vs never/former) cigarette smoking among adults aged ≥40 years at Wave 5 (2018–2019) of the Population Assessment of Tobacco and Health (PATH) Study

*Characteristics*	*Model 1 (N=11192)*	*Model 2 (N=10952)*	*Model 3 (N=10950)*
	*RR (95% CI)*	*ARR (95% CI)*	
**Geographical location**			
Urban ®	1	1	1
Suburban	**0.83 (0.74–0.92)**	0.97 (0.87–1.09)	1.00 (0.90–1.10)
Town	1.15 (0.98–1.34)	1.07 (0.91–1.25)	1.00 (0.87–1.15)
Rural	**1.17 (1.03–1.32)**	**1.14 (1.01–1.29)**	1.08 (0.96–1.21)
**Mean cigarette risk perception scale** (4 items)*			**2.05 (1.94–2.18)**

Model 1 was unadjusted. Model 2 was adjusted for sociodemographic characteristics (age, sex, race, education level, and household income). Model 3 was adjusted for sociodemographic characteristics and cigarette risk perceptions.

* Mean across four cigarette risk perceptions items, with higher values indicating a lower perception of risk (range: 1–5). RR: risk ratio. ARR: adjusted risk ratio. The ARR represents the increased adjusted relative risk per 1 unit increase in the scale. Bold values denote statistical significance, p<0.05.

® Reference category.

### Sensitivity analysis

Overall, adults who currently (vs never or formerly) smoked cigarettes perceived cigarettes as being less harmful regardless of geographical location (overall mean=1.33 for never/former and 1.82 for current) (Supplementary file Table 1). Among adults who reported never/former smoking, risk perceptions were similar for those in rural (mean=1.33) and urban (mean=1.32) locations. However, among adults who reported current smoking, those in rural areas perceived cigarettes as less harmful (mean=1.91) than those in urban areas (mean=1.78). For the individual scale items, risk perceptions were similar between adults in urban and non-urban areas who reported never/former smoking. However, among adults who currently smoked, respondents in rural areas strongly agreed or agreed with beliefs about cigarette health risks less often than respondents in urban areas. The largest difference was observed for the statement ‘smoking can cause lung disease in non-smokers from secondhand smoke’, with which 73.6% of urban versus 63.4% of rural residents strongly agreed/agreed. A similar trend was observed for ‘smoking can cause lung cancer in smokers’ (90.2% vs 85.6% strongly agreed/agreed for urban and rural, respectively). However, the perception that ‘smoking can cause lung disease in smokers’ (92.3% vs 89.3% strongly agreed/agreed for urban and rural, respectively) and reporting that cigarettes are very/extremely harmful to health (69.0% vs 66.2%, respectively) were similar for urban and rural respondents (Supplementary file Table 1).

Shown in Table 3, across the four individual items of perceptions (Models 3a– 3d), two individual items confounded the rural-current smoking association whereas two did not. Specifically, believing that cigarettes are ‘Harmful’ (vs ‘Not harmful’) to health and responding ‘Agree’ (vs ‘Not agree’) to the statement that secondhand smoke causes lung disease in people who do not smoke, confounded the rural association with current smoking. Responding ‘Agree’ (vs ‘Not agree’) to statements about smoking causing lung cancer or lung disease in people who smoke, did not confound the association. In addition, the largest ARR for risk perceptions on current smoking was observed for ‘how harmful do you think cigarettes are to health?’ (ARR=3.02; 95% CI: 2.81–3.26), which was significantly larger than the ARRs for ‘smoking can cause lung cancer in smokers’ (ARR=2.46; 95% CI: 2.16–2.80), ‘smoking can cause lung disease in smokers’ (ARR=2.17; 95% CI: 1.93–2.44), and ‘smoking can cause lung disease in non-smokers’ (ARR=2.57; 95% CI: 2.39–2.77). For each of these associations, a lower perception of risk was significantly positively associated with current smoking independent of other factors.

**Table 3:** Sensitivity analysis: multivariable regressions examining associations between geographical location, individual cigarette risk perceptions, and current (vs never/former) cigarette smoking among adults aged ≥40 years at Wave 5 (2018-2019) of the Population Assessment of Tobacco and Health (PATH) Study

*Characteristics*	Model 3a* (N=10876)	Model 3b* (N=10902)	Model 3c* (N=10895)	Model 3d* (N=10934)
	*ARR (95% CI)*			
**Geographical location**				
Urban ®	1	1	1	1
Suburban	0.99 (0.89–1.10)	0.98 (0.88–1.09)	0.98 (0.88–1.08)	0.99 (0.89–1.09)
Town	1.07 (0.92–1.26)	1.06 (0.91–1.24)	1.03 (0.88–1.19)	1.02 (0.88–1.18)
Rural	**1.13 (1.01–1.27)**	**1.14 (1.01–1.28)**	1.10 (0.98–1.23)	1.11 (0.99–1.24)
**Smoking can cause lung cancer in smokers^§^**				
Not agree	**2.46 (2.16–2.80)**			
**Smoking can cause lung disease in smokers^§^**				
Not agree		**2.17 (1.93–2.44)**		
**Smoking can cause lung disease in non-smokers^§^**				
Not agree			**2.57 (2.39–2.77)**	
**How harmful are cigarettes to health^†^**				
Not harmful				**3.02 (2.81–3.26)**

* ARR: adjusted risk ratio; adjusted for age, sex, race, education level, and household income.

§ Reference category is ‘Agree’ which combines strongly agree and agree. ‘Not agree’ combined neither, disagree, and strongly disagree.

† Reference category is ‘Harmful’ which combines very and extremely harmful. ‘Not harmful’ combines slightly, somewhat, and not at all harmful. Bold values denote statistical significance, p<0.05.

® Reference category.

## DISCUSSION

In this large nationally representative study of US adults aged ≥40 years, rural (vs urban) residence was significantly positively associated with current smoking independent of sociodemographic covariates (including income and education). After accounting for cigarette risk perceptions in the model, the rural-current smoking association was attenuated, suggesting that lower perceptions of risk among adults who reside in rural versus urban locations of the US play an important role in current smoking prevalence disparities. This finding is consistent with prior studies indicating that non-urban residence is associated with higher rates of tobacco use among youth and adults^3-5^ and extends such work by showing a significant association between rural residence and current smoking among a sample of older US adults who are at greatest risk for developing smoking-related lung disease^26^. Moreover, the association between rural (vs urban) residence and current smoking was accounted for by differences in cigarette health risk perceptions.

We found that overall risk perceptions were similar among adults in rural and urban areas if they reported never or former smoking; however, adults in rural (vs urban) areas perceived cigarettes as being less harmful to health if they currently smoked, and these differences were large enough to confound the association between rural residence and current smoking. We also observed some differences for the individual risk perceptions items among adults who reported current smoking, which are consistent with results from our sensitivity analyses. Specifically, among adults who currently smoked, those in rural areas agreed with the statement that ‘smoking can cause lung disease in non-smokers from secondhand smoke’ and reported cigarettes to be extremely or very harmful to health less often than those in urban areas. The biggest discrepancy was for the item related to beliefs about the harms of secondhand smoke. Despite research showing that secondhand smoke exposure is an independent risk factor for COPD and lung cancer among adults who have never smoked cigarettes^27,28^, smoke-free policies are less common in rural areas of the US^29,30^. Even when smoke-free policies for public outdoor or residential areas are present, noncompliance is high in rural areas^29-31^. Our findings suggest a potential avenue for communication campaigns aimed at increasing risk perceptions surrounding secondhand smoke exposure specifically that in turn, may reduce cigarette smoking among older adults living in rural areas. Effective communication campaigns may need to be tailored specifically to those who live in rural communities^32,33^.

### Limitations

Our study has several strengths, including use of a nationally representative sample of a group at high risk for tobacco-related diseases and use of a newly released (in July 2024) PATH Study geographical location variable. However, this study also has limitations. First, there are other factors that put people in rural areas of the US at increased risk for tobacco use (e.g. lower tobacco excise taxes^34^; greater social norms around tobacco^35^), many of which were not measured or included in this study. It is therefore possible that the associations found in our study could be accounted for by unmeasured factors and residual confounding. Second, causality cannot be determined based on our cross-sectional study. Rigorous longitudinal studies that examine changes in respondents’ geographical location and how it might be associated with changes in cigarette risk perceptions or smoking status are needed. Finally, we intentionally limited the analytic sample to US adults aged ≥40 years given that this subpopulation is at greatest risk for developing smoking-related lung disease; findings may not generalize to younger populations or older adults in other countries. Future work could also consider examining interactions between geographical location and other sociodemographic characteristics (e.g. sex) to determine whether geographical location has a larger association with current smoking for specific sociodemographic groups.

## CONCLUSIONS

In this large nationally representative study, the association between rural (vs urban) residence and current smoking was accounted for by differences in cigarette risk perceptions among a sample of older (aged ≥40 years) adults who are at greatest risk of developing tobacco-related lung disease. Among adults who currently smoked, those in rural areas agreed with statements about specific cigarette health risks (e.g. risk of lung disease from exposure to secondhand smoke) less often than those in urban areas, highlighting potential targets for communication campaigns. More work is needed on intersecting factors promoting tobacco use among people in rural areas and how to best promote tobacco cessation among older adults.

## Supplementary Material



## Data Availability

The data supporting this research are available from the following source: https://www.icpsr.umich.edu/web/NAHDAP/studies/36231
