# Supplementary material for: Geographical location, cigarette risk perceptions, and current smoking among older US adults
Source: Tob Induc Dis. 2024 Sep 12;22:10.18332/tid/191827. doi: 10.18332/tid/191827 (PMC11391726; doi:10.18332/tid/191827)
Supplement: Supplementary file 1 [file TID-22-158-s1.pdf]

Supplemental Table 1. Cigarette risk perceptions overall, by urban/non-urban residence, and by current (vs never/former) smoking status among adults aged 40+ years at Wave 5 (2018-2019) of the Population Assessment of Tobacco and Health (PATH) Study, N=11,192 who reported on their smoking status at Wave 5.

	**Never or former smoking N=7,059**				**Current smoking N=4,133**			
	**Urban N=2,098**	**Suburban N=2,652**	**Town N=626**	**Rural N=1,683**	**Urban N=1,255**	**Suburban N=1,229**	**Town N=477**	**Rural N=1,172**
	**Weighted Column % (95% confidence interval)**							
**Weighted mean (SD) cigarette risk perceptions[1]**	1.32 (0.43)	1.28 (0.36)	1.37 (0.46)	1.33 (0.41)	1.78 (1.04)	1.76 (0.94)	1.84 (1.06)	1.91 (1.06)
**Based on what you believe, how much do you agree or disagree with the following statements. Smoking can cause…**								
**Lung cancer in smokers**								
Strongly agree or Agree	97.0 (95.1-98.2)	98.6 (97.9, 99.0)	97.8 (95.8-98.9)	98.4 (97.7-98.9)	90.2 (88.1-91.9)	91.3 (89.1-93.0)	88.6 (85.9-90.9)	85.6 (83.1-87.8)
Neither, Disagree, or Strongly disagree	3.0 (1.8-4.9)	1.4 (1.0-2.1)	2.2 (1.1-4.2)	1.6 (1.1-2.3)	9.8 (8.1-11.9)	8.7 (7.0-10.9)	11.4 (9.1-14.1)	14.4 (12.2-16.9)
**Lung disease (such as emphysema) in smokers**								
Strongly agree or Agree	97.4 (95.9-98.4)	98.7 (98.2-99.1)	97.4 (94.8-98.7)	99.0 (98.3-99.3)	92.3 (90.6-93.8)	93.1 (91.4-94.5)	90.8 (88.0-93.0)	89.3 (87.0-91.2)
Neither, Disagree, or Strongly disagree	2.6 (1.6-4.1)	1.3 (0.9-1.8)	2.6 (1.3-5.2)	1.0 (0.7-1.7)	7.7 (6.2-9.4)	6.9 (5.5-8.6)	9.2 (7.0-12.0)	10.7 (8.8-13.0)

**Lung disease in non-smokers from second-hand smoke**								
Strongly agree or Agree	91.8 (90.4-93.0)	92.8 (91.4-94.1)	88.4 (84.8-91.2)	91.1 (89.5-92.4)	73.6 (70.7-76.3)	70.7 (67.5-73.7)	71.6 (68.2-74.8)	63.4 (60.2-66.4)
Neither, Disagree, or Strongly disagree	8.2 (7.0-9.6)	7.2 (5.9-8.6)	11.6 (8.8-15.2)	8.9 (7.6-10.5)	26.4 (23.7-29.3)	29.3 (26.3-32.5)	28.4 (25.2-31.8)	36.6 (28.9-32.1)
**How harmful do you think cigarettes are to health?**								
Not at all, slightly, or somewhat harmful	5.1 (3.9-6.6)	4.4 (3.5-5.4)	6.9 (4.6-10.1)	5.5 (4.5-6.6)	31.0 (27.9-34.2)	28.5 (25.8-31.4)	35.7 (31.6-40.1)	33.8 (30.7-37.0)
Very or extremely harmful	94.9 (93.4-96.1)	95.6 (94.6-96.5)	93.1 (89.9-95.4)	94.5 (93.4-95.5)	69.0 (65.8-72.1)	71.5 (68.6-74.2)	64.3 (59.9-68.4)	66.2 (63.0-69.3)

[1]The four separate cigarette risk perceptions items (labeled item 1-item 4) were reverse coded as needed such that across all four items, higher values indicated lower perceptions of risk. Then, an average risk perception score across all four items was calculated for each respondent.

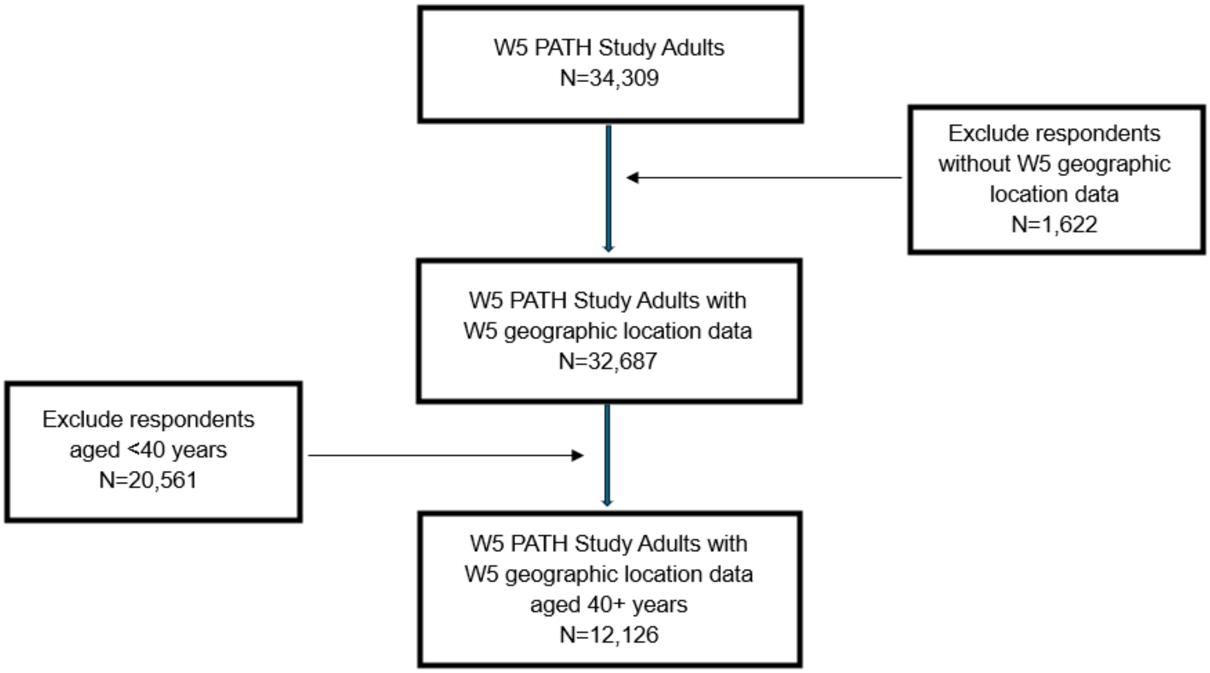

Supplemental Figure 1. Flow diagram of study participation, Wave 5 of the Population Assessment of Tobacco and Health (PATH) Study, 2018-2019.

© 2024 Ozga J.E. et al.
